# Rapid surface uplift and crustal flow in the Central Andes (southern Peru) controlled by lithospheric drip dynamics

**DOI:** 10.1038/s41598-022-08629-8

**Published:** 2022-04-01

**Authors:** Oğuz H. Göğüş, Kurt Sundell, Ebru Şengül Uluocak, Joel Saylor, Uğurcan Çetiner

**Affiliations:** 1 Eurasia Institute of Earth Sciences, Istanbul Technical University (ITU), İstanbul, Turkey; 2 Department of Geosciences, Idaho State University, Pocatello, USA; 3 Department of Geophysical Engineering, Çanakkale Onsekiz Mart University, Çanakkale, Turkey; 4 Department of Earth, Ocean and Atmospheric Sciences, University of British Columbia, Vancouver, Canada

**Keywords:** Solid Earth sciences, Geodynamics

## Abstract

The high flux magmatism, crustal shortening/extension and plateau formation in Cordilleran orogenic systems have been explained by removal of lithosphere (lower crust and the sub-arc mantle lithosphere) that develops beneath the magmatic arc and hinterland regions. However, the primary role of this process driving surface uplift, and crustal deformation is not well understood. Here, reconciling geodynamic model predictions with lithospheric structure and paleoelevation estimates, we suggest that viscous drip-type lithospheric removal from beneath the Central (Peruvian) Andes can explain several tectonic features: (1) “double humped” shaped/axisymmetric topographic profile and rapid surface rise (up to 1.2 km in ~ 4.31 Myrs); (2) thicker crust associated with the lower surface elevation of the Altiplano plateau (Lake Titicaca region) (negative residual topography) and higher topography and thinner crust of Western and Eastern Cordilleras (positive residual topography); and (3) faster wave speed (colder)/sub-Moho anomaly underlying the Altiplano, surrounded by slower speed anomalies on both western arc-forearc areas and parts of the eastern Cordillera and Sub-Andes. Our results emphasize the important role of lithospheric drip and associated mantle dynamics in the transient evolution of Andean orogeny controlling surface uplift and crustal flow and thickening.

## Introduction

The Central Andean Plateau (Altiplano-Puna region) of the Nazca-South America subduction system^1–6^ is the second highest orogenic plateau after Tibet and its evolution has been a topic of growing interest in wide range of scientific disciplines, including geosciences, atmospheric and ocean sciences^7–11^. Compelling geological, geophysical and petrological evidence are used to infer that lithospheric removal/thinning and crustal shortening/thickening has occurred in the central Andes, however, the geodynamic controls of these processes on plateau formation remains enigmatic. By integrating deep lithospheric and crustal structure with uplift histories, the subject of this work is to show that the origin of the (rapid) plateau uplift, as invoked by paleoelevation data, and the inferred lower crustal flow, as well as anomalous topography in the Peruvian Central Andes/northern Altiplano is at least in part controlled by the convective removal (viscous drip type) of the lithosphere in this part of the Andean orogenic system.

**Figure 1:**
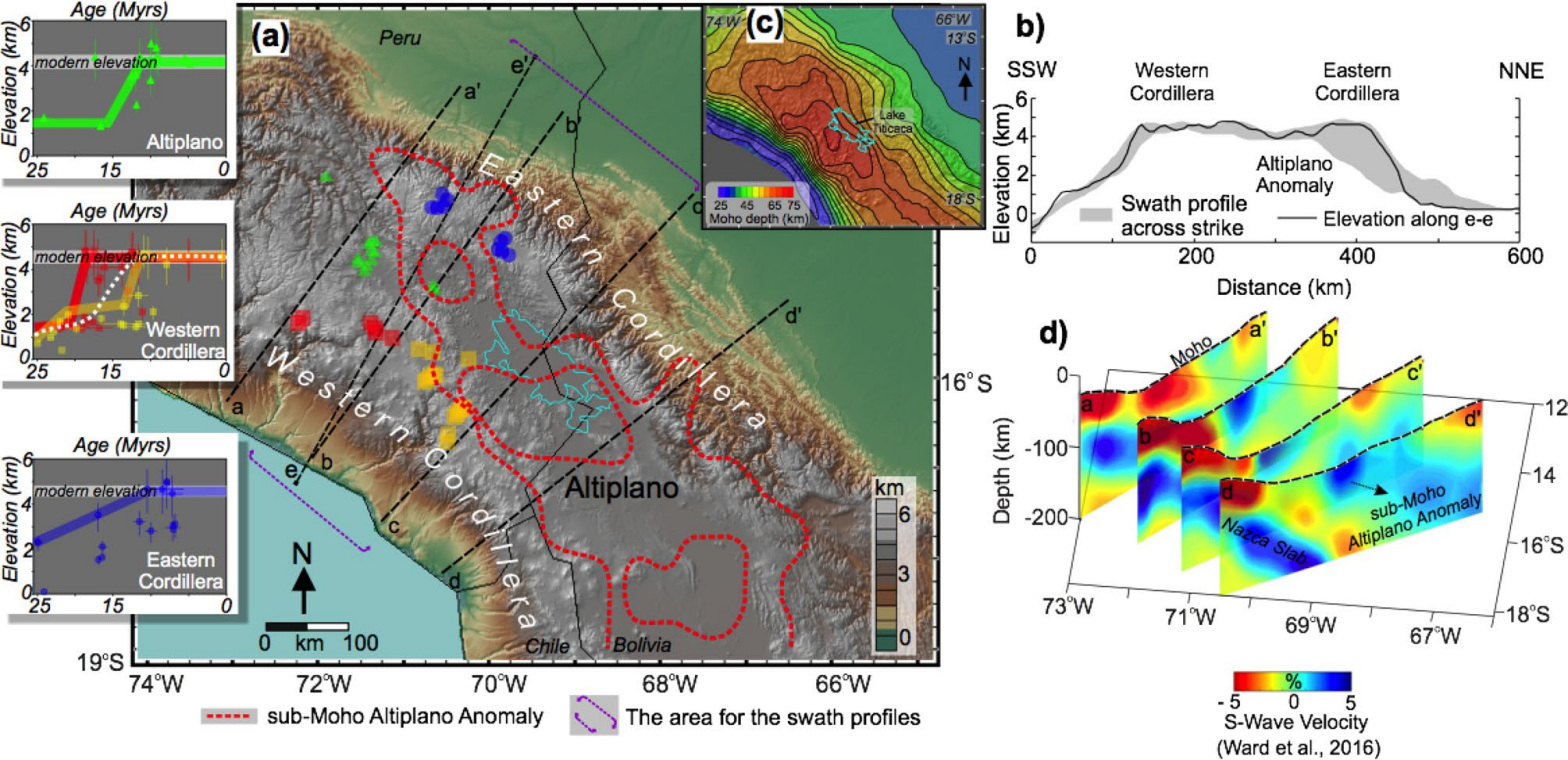
(**a**) Peruvian part of the Central Andean Plateau with topographic variations, including sub-Moho Altiplano Anomaly (thick dashed red line indicates the edge of fast seismic wave speed anomaly^18,19^). Base map made with GeoMapApp (http://www.geomapapp.org)^20^. Diagrams in the left edge of the map show the paleoelevation calculations (25–0 Ma) for Altiplano, Eastern and Western Cordillera^14^. While available paleoelevation estimates across the Peruvian Central Andes show spatial and temporal variations and a range of uplift amounts, the work by Sundell et al. collectively interprets the independent geological information, other published paleolevation and isotopic data to better approximate the topographic evolution of the region within the uncertainty of ~ 500 m. For example, there is estimated ~ 2 km of elevation gain in the Altiplano between 15 and 10 Ma (**b**) Topographic swath profile indicating minimum and maximum elevations for the area in (**a**). Black line shows the topographic cross-section along (**e**–**e′**). (**c**) Map of Moho depth^18^. (**d**) Four lithospheric-upper mantle scale cross sections perpendicular to the strike of the orogen, produced from seismic tomography models (S wave velocity)^18^ (please see the map for their locations). Note the sub-moho Altiplano (higher speed) anomaly hanging in the middle of the profile. (**c**, **d**) is created through Matlab software version R2016b (https://www.mathworks.com/).

The central Andes is characterized by the low-relief internally drained Altiplano plateau with ~ 4 km surface elevation above sea level where it is bounded by the Western Cordillera magmatic arc and the Eastern Cordillera fold-thrust belt, associated with 4–6 km high elevations (Fig. 1a,b). Paleoelevation records based on variety of techniques document multiple spatially limited phases of relatively rapid uplift of the Central Andes (i.e., up to 1 km elevation increase in 1 Myr)^12–14^. Sundell et al.^14^ recently integrated regional paleoelevation data with independent geological interpretations to document significant elevation gain between ~ 25 and 10 Ma in the Peruvian Central Andes (see plots in Fig. 1a). The paleoaltimetry records also show that both the Peruvian Western and the Eastern Cordilleras underwent earlier stages of uplift compared to the northern Altiplano (e.g., 20–15 Ma). Specifically, surface uplift of 1.5–2.5 km in the Altiplano and southern Peruvian Western Cordillera (at 15.5° S–17° S) post-dated 15 Ma^14–16^. In contrast, protracted surface uplift of 1.5–2 km in the Eastern Cordillera occurred over ~ 15 Myr starting from ~ 2 km at 25 Ma. Furthermore, uplift in the central Western Cordillera (15° S–15.5° S) also preceded 15 Ma, with stable isotopic data indicating rapid surface uplift of 2–2.5 km from 20 to 17 Ma^14,17^.

The tectonic evolution of the Central Andes since Eocene has largely been accounted for orogen-wide shortening and thickening from the Western Cordilleran magmatic arc, across the Altiplano to Eastern fold-thrust belt^3,4,21–24^ within the large-scale hinterland-foreland basin system^25–28^ and as a result of flat slab subduction^29,30^. However, especially for the northern part of the Central Andes of southern Peru, crustal shortening estimates based on kinematic reconstructions cannot justify the present day crustal thickness variations^22,31–33^, which implies that Altiplano uplift to high topography may be driven by other geological processes, such as lower crustal flow^34,35^ or magmatic addition^36^. Furthermore, over the entire Andes (Altiplano-Puna) the rapid development of topographic transients (e.g., subsidence or uplift) have also been interpreted to be the result of lithospheric removal rather than solely to plate shortening^24,37–44^, yet the impact of such lithospheric scale dynamic process(es) and their geological responses are not well understood in terms of magnitude, timing, scale, and pattern, in the course the Andean orogenic evolution.

Recent seismic (S wave) tomography model has shown a zone of anomalously fast lithospheric mantle beneath the Altiplano of Peru/northern Bolivia^18^. The Altiplano maintains a relatively lower elevation despite being above the thickest crust in the Andes (Fig. 1c,d). In contrast, the higher elevation Eastern and Western cordilleras are underlain by thinner crust but also relatively slower seismic wave speed (e.g. warmer temperature) upper mantle. Further, Ward et al^18^ suggests that the Altiplano is 1–2 km lower than would be predicted by Airy isostatic considerations of the crust and attribute the difference to the presence of the denser sub-crustal/mantle lithosphere underlying the crust.

In this work we investigate the extent and magnitude to which viscous dripping of the lithospheric root and subsequent mantle uprising controls the plateau uplift in the Peruvian section of the Central Andes using numerical geodynamic experiments. Model predictions satisfactorily explain the nearly axisymmetric pattern of surface uplift with higher surface elevation of the Western and Eastern cordilleras and relatively lower elevation in the Altiplano associated with thicker crust and high seismic wave speed sub-Moho anomaly interpreted from seismic tomography model^18^. Our results have implications for understanding the role of lithospheric drips that presumably control the rapid surface uplift (10 and 6 Ma) in Bolivian Altiplano^37^, as well as Miocene Arizaro basin in Puna Plateau^41^ and other parts of the Andean orogenic system.

## Results

**Figure 2:**
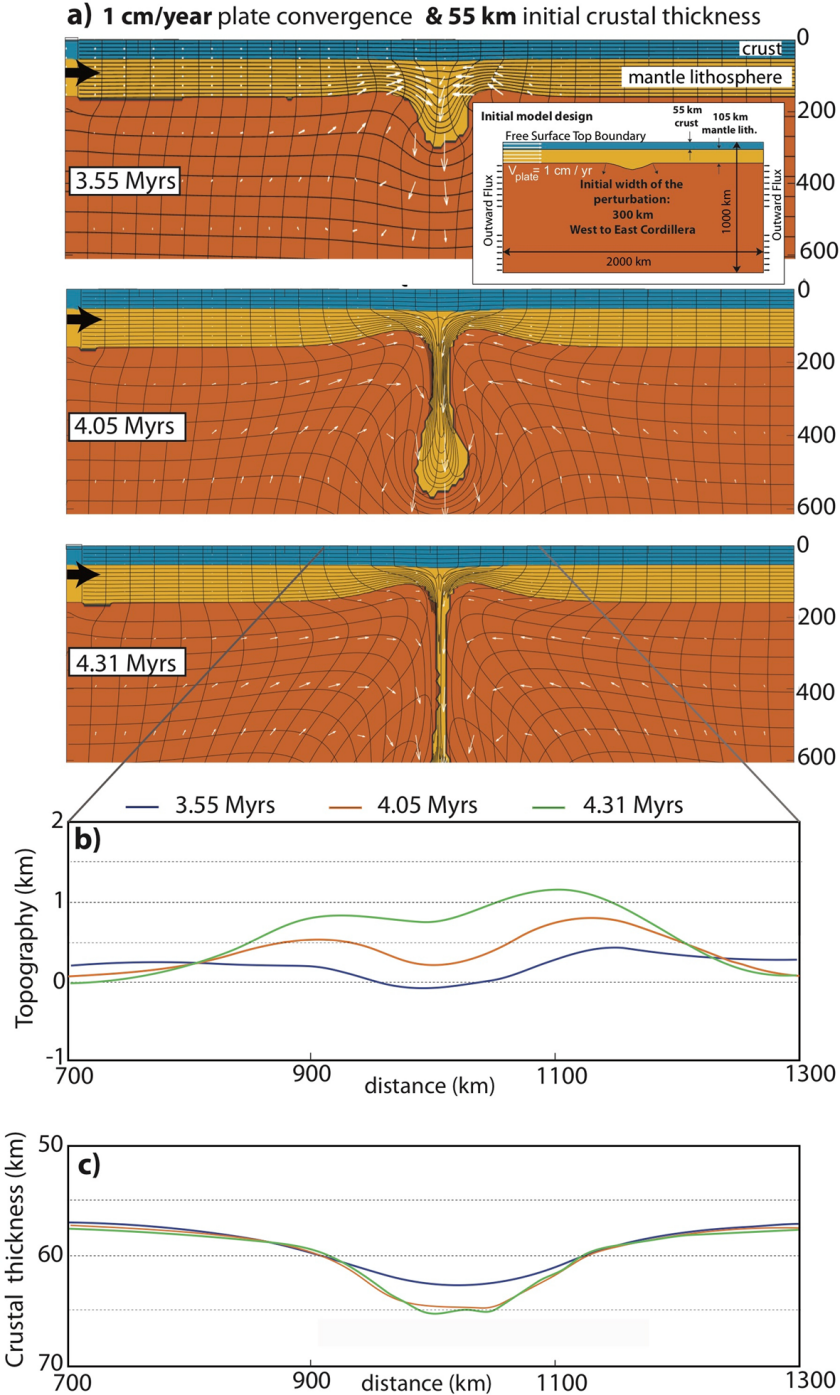
(**a**) Evolution of lithospheric (viscous drip type) removal (reference/preferred) model for timeframes 3.55 Myrs, 4.05 Myrs and 4.31 Myrs (approximately) corresponding to 10 Ma for the Peruvian part of the Central Andean Plateau. (**b**) Topographic evolution, (**c**) Crustal thickness variations for each timeframes shown in a. Plate convergence velocity Vp = 1 cm/year is applied on the left boundary of the model domain. The velocity boundary conditions are described as a constant inflow from the top to 160 km depth at the left boundary (LAB) with constant outflow imposed from beneath the lithosphere down to 1000 km depth through both sides. The magnitude of the outflow equals the volume of inflow to conserve the mass in the model domain. The lithosphere on the right margin is held fixed (pinned) for all model experiments.

We use 2-D thermo-mechanical numerical experiments to explore the development drip type lithospheric removal as well as its transient topographic and crustal consequences, applicable to Central Andes. The rheological characteristics of each layer used in this work (e.g., crust, asthenosphere), are provided in Table 1 and general model properties (e.g., geothermal gradient, initial densities, layer thicknesses), and descriptions of the numerical code (SOPALE) are given under Methods section. Inset frame in Fig. 2a shows the initial model design for lithospheric dynamics and the geometrical characteristics implemented in the model.

**Table 1:** Rheological parameters for numerical models: dry olivine mantle^45^, wet quartzite crust^46^.

	Parameter	Continental crust	Sub-arc mantle lithosphere	Sub-lithospheric mantle
A	Viscosity parameter	1.1 × 10^28^ Pa^−4^*/s*	10^38^ Pa^−n^/s	4.89 × 10^17^ Pa^−3.5^/s
*n*	Power exponent	4.0	3.5	3.5
Q	Activation energy	223 kJ mol^−1^	0	535 kJ mol^−1^
*ϕ*	Effective internal angle of friction	15°–2°	0	0
ρ_o_	Reference density	2840 kg m^−3^	3300 kg m^−3^	3260 kg m^−3^
σ_y_	Plastic yield stress	1 MPa	0	0
α	Coefficient of thermal expansion	2.0 × 10^–5^ K^−1^	2.0 × 10^–5^ K^−1^	2.0 × 10^–5^ K^−1^

We first show the behavior of a reference/preferred model (Model 1 in Fig. 2) that has boundary conditions (Vp = 1 cm/year) approximately estimated for this part of the Central Andean orogenic system as the plate (lithospheric) shortening/convergence is controlled by the subduction of the Nazca plate beneath the South America. For alternative models, the response of the model results to higher plate shortening magnitude (Model 2) and the lesser (40 km) initial crustal thickness (Model 3) is explored, and finally the results are reconciled with observations from the northern part (Peruvian) of the Central Andes.

### Central Andean lithospheric drip/downwelling

A representative reference model for the tectonic evolution of southern Peru at ~ 15–10 Ma is shown in Fig. 2a. Here, Vp = 1 cm/year plate convergence velocity is imposed on the left margin of the model and the initial thickness of the crust is 55 km. By 3.55 Myrs after the experiment begins the concave viscous bulging of the lithospheric root (gravitationally unstable) grows as the instability starts to descend into the asthenospheric mantle. The displacement vectors associated with the tracking particles in the numerical formulations suggest that such instability (drip) pulls material from both sides driving crustal and (sub-crustal) lithospheric thickening. Meanwhile, lithosphere is attenuated in regions peripheral to the instability as hot and buoyant asthenospheric material is advected upward and ascends in into the regions of thinned mantle lithosphere. The development of this viscous dripping lithosphere process is rapid, and by 4.05 Myrs the root/head of the instability sinks as deep as ~ 600 km. There is significant (approximately 400 km) vertical stretching/thinning of the lithosphere by 4.31 Myrs due to the downward pull exerted by the falling drip. During downwelling process, the convective mantle return flow develops over the stripe of the lithosphere, as it is recycled into the mantle. The asthenospheric upwelling/mantle replacement area widens as more viscous lithosphere has been removed from beneath the overlying crust. Although it is thinned significantly, by this time, the upper part of the lithospheric piece remains still attached to the crust as the removal process continues^47^.

### Rapid surface uplift and the viscous coupling between the crust and mantle lithosphere

Figure 2b shows that a basin develops (< 0 km elevation) in the centre where the crust is forced down by the sinking lithosphere (3.55 Myrs). Concurrently, lithospheric removal causes uprising of the buoyant mantle under the crust resulting in topographic highs (> 0 km elevation) adjacent to the basin. Such (nearly) axisymmetric surface elevation pattern (e.g., central subsidence and adjacent uplifts) continues by 4.05 Myrs with increasing magnitudes (> 500 m at x = 900 and x = 1100 km) while topographic wavelength over the downwelling has slightly diminished (less broad). The decrease in topographic wavelength is because the width of the sinking lithosphere in the upper asthenosphere (< 200 km depth) has decreased through necking (Fig. 2a) and the resulting surface deflection becomes more localized.

By 4.31 Myrs, the overall axisymmetric topographic profile, characteristic to this viscous drip type lithospheric removal has slightly been distorted since plate shortening is more prevalent due to the increased plate convergence imposed from the left side of the model. Nevertheless, the approximately “double humped” surface elevation (viz. higher on the sides, lower in the centre) still persists. The average surface topography is less than 1 km except over the zone of 100 km (x = 1050–1150 km). While the dripping and sinking lithosphere and the related mantle dynamics control rapid evolution of the plateau uplift, the upper part of the instability is yet still attached to overlying crust. This is similar to the persistence of a sub-crustal anomaly shown in the lithospheric structure inferred by seismic tomography models^18^ (see section for discussion on this). Such viscous coupling between crust and the lithospheric drip results in continued flow of the lower crust from the margins towards the centre where the crust becomes 65 km thick (Fig. 2c) (Please also see Supplementary Fig. 1 for the viscosity variation across the central section of crust-mantle interface pertinent to each model timeframe). Hence, the margins are characterized by thinner crust (60 km). Here, the lower elevation in the centre is underlain by the thicker crust (internally drained *Altiplano*), whereas the sides that have higher elevation (*Western and Eastern Cordilleras*) are associated with thinner crust and supported by asthenospheric (buoyant) mantle. This is paradoxic to Airy type crustal isostatic compensation where higher surface elevation is compensated by thicker crust when the densities between the compared regions are approximately equal.

### Controls of model parameters on the evolution of lithosphere in the Central Andes

**Figure 3:**
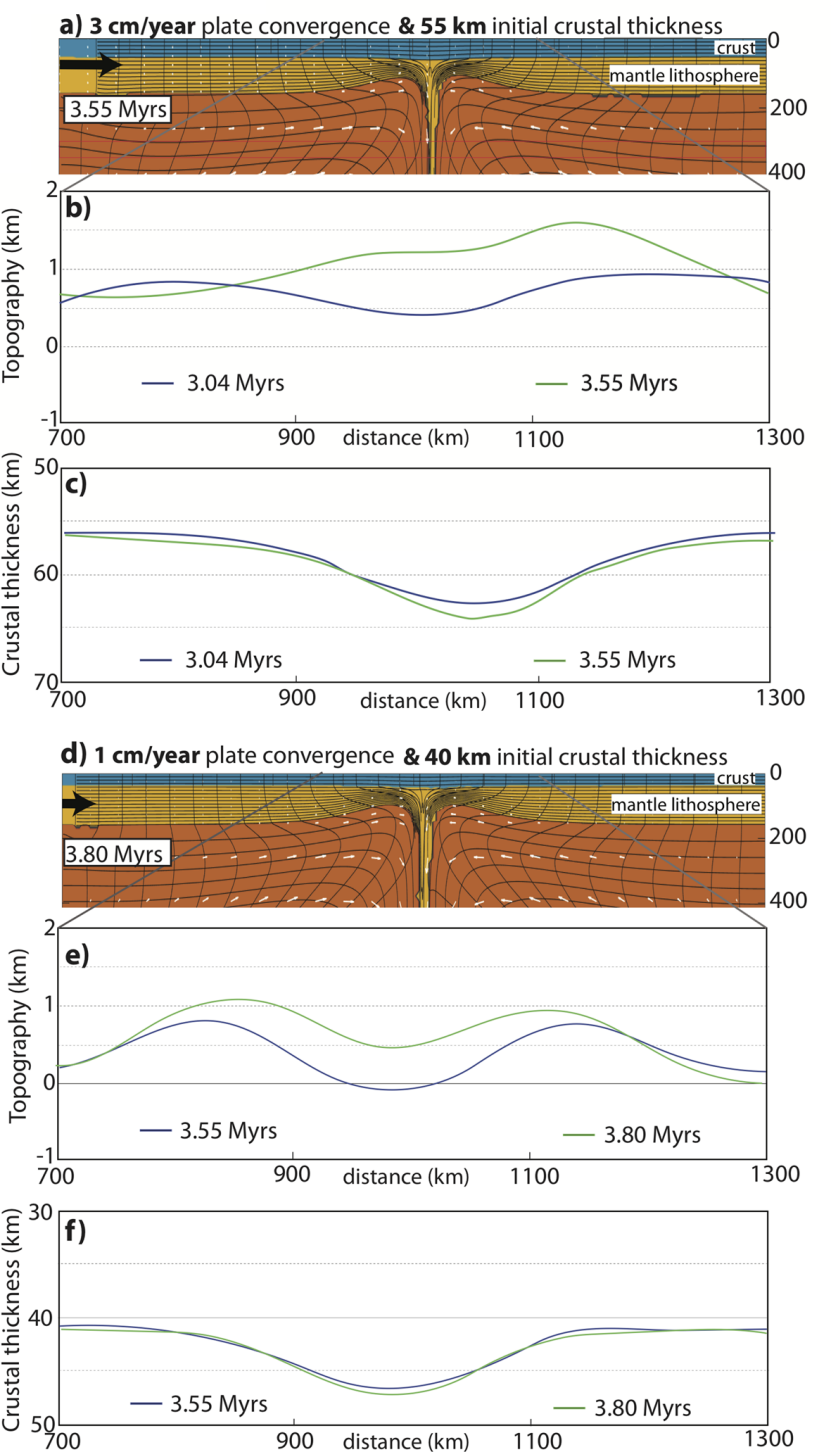
(**a**) Geodynamic configuration of lithospheric removal model for timeframe 3.55 Myrs. Plate convergence velocity Vp = 3 cm/year is applied on the left boundary of the model domain. (**b**) Topographic evolution for timeframes 3.04 and 3.55 Myrs, (**c**) Crustal thickness variations for each timeframes shown in (**b**). (**d**) Geodynamic configuration of lithospheric removal model for timeframes 3.80 Myrs. Plate convergence velocity Vp = 1 cm/year is applied on the left boundary of the model domain but the initial crustal thickness is reduced to 41.5 km (approximately global average). (**e**) Topographic evolution for timeframes 3.55 and 3.80 Myrs, (**f**) Crustal thickness variations for each timeframes shown in (**b**).

While the predicted strain across the Central Andes is predominantly contemporaneous^4,48^, the estimates for the amount of E–W shortening vary along the strike of the orogen. For example, the estimated shortening is 250–300 km in the central (Bolivia, 20° S) and 50–120 km in the north (Peru, 13° S) and in the south (Argentina, 27° S)^5,32,33,49^. Based on such variability of shortening across the Andes, we test how the change in the magnitude of plate shortening (imposed plate convergence velocity) controls the evolution of the lithospheric dripping process and the amount of variation in surface topography and crustal thickness. Here, all other experimental parameters are kept same with Model 1, described above. When plate convergence is removed (Vp = 0), the gravitational instability development process becomes slower approximately 1 Myr. The results of this experiment are not shown because they are quite comparable to the Model 1 (e.g. negligible tectonic effect). However, Model 2 with faster plate convergence velocity (Vp = 3 cm/year) results in more rapid descent of the lithospheric root (Fig. 3a) and show marked differences in terms of elevation gain and topographic pattern. Namely, by 3.55 Myrs there is approximately similar amount of lithospheric removal as well as vertical extension of the sinking lithosphere and the depth position of it with that of 4.31 Myrs in Model 1. Although the topographic profile is symmetrical during the early stages of the model development (e.g. 3.04 Myrs), by 3.55 Myrs it becomes distinctly asymmetric where the peak on the right side exceeds > 1.5 km and another one (on the left) is above 1 km, showing decreasing trend of elevation to the left (x = 700 km) (Fig. 3b,c).

The geodynamic evolution of the lithospheric drips is controlled by the buoyancy of the crust and the underlying sub-arc mantle lithosphere^50–52^. In the following experiment, while keeping all other model parameters same with Model 1, we set the initial thickness of the crust to 41.5 km (approximately global average) as well as the approximate thickness of the foreland of the Andean Plateau. With relatively thinner crust, the thickness of the gravitationally unstable layer is now 15 km thicker (lithospheric thickness has been kept same with that of other models) and as a result, the descent rate of lithospheric drip has been increased compared to Model 1 (Fig. 3d). Notably, the symmetrical pattern of topographic profile is not distorted during the model evolution (3.55 and 3.80 Myrs) because the dynamic effects associated with the downwelling lithosphere (drip pull forces) are enhanced relative to crustal shortening via plate convergence. As such, the pull force exerted by the instability is amplified with thicker/negatively buoyant mantle lithosphere. The amplitude of uplift in the interior is ~ 500 m less compared to maximum 1 km high flanks (Fig. 3e), diagnostic to instability induced “double humped”-shaped topographic profile. In the centre, the crust is thickened to more than 45 km owing to the viscous drag induced by the downwelling where the surface topography is lower compared to the flanking peaks (Fig. 3f).

## Discussion

**Figure 4:**
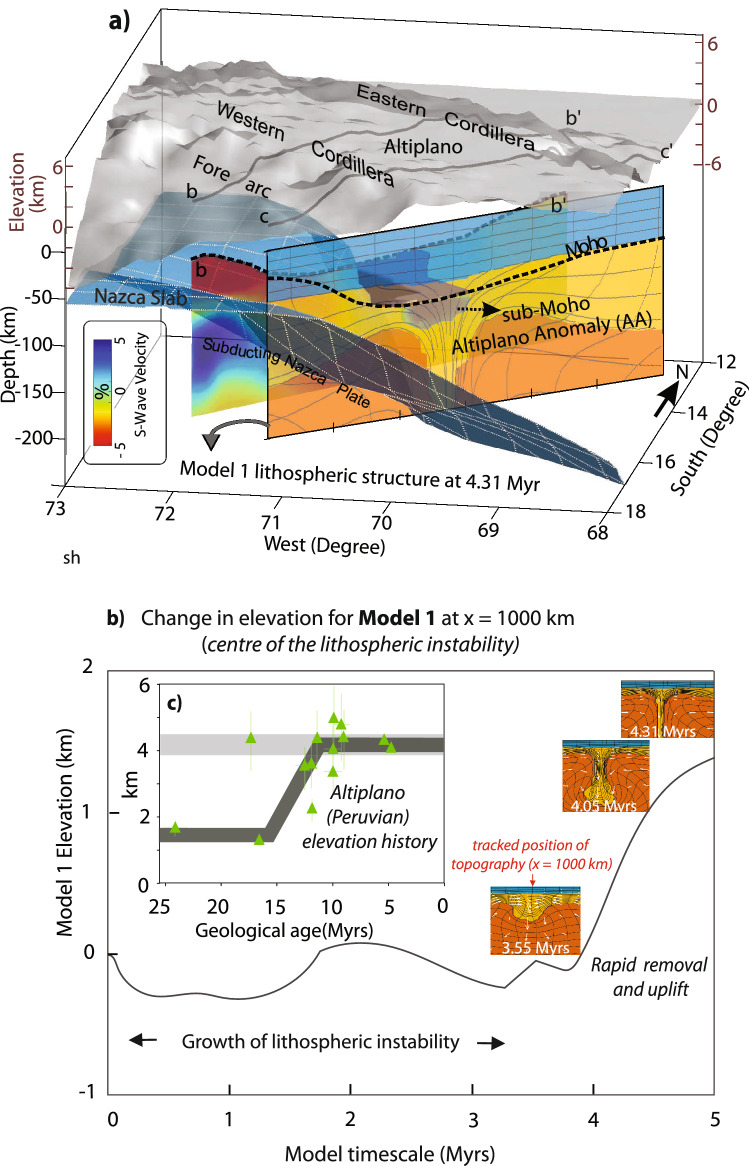
(**a**) S-wave velocity distributions across Peruvian part of Central Andes^18^ where high seismic wave speed (sub-Moho Altiplano anomaly) is shown over two cross sections (**b**–**b′** and **c**–**c′**). Numerical model result (Model 1) for 4.31 Myrs is reconciled with the inferred lithospheric structure derived from seismic analyses. The Moho (from Fig. 1c) in all cross-sections is shown as a dashed black line, isosurface of the subducting Nazca Slab is derived from the Slab 1.0^60^. Figure is created through Matlab software version R2016b (https://www.mathworks.com/), (**b**) Predicted trajectory of topography for Model 1 above the lithospheric drip (x = 1000 km), (**c**) Paleoelevation estimates of the Altiplano region compilted from^14^.

The geodynamic model results presented above are consistent with seismological studies that document regions beneath the Central Andes where low seismic wave speed (hotter) anomalies at shallow mantle depths are juxtaposed against high speed (colder) anomalies^53–58^ due to the removal of (possibly eclogitized) lower crust and mantle lithosphere in the Central Andes^16,17,59^. More recent seismic surface wave tomography models, imaging the lithospheric characteristics in the southern Peru/northern Altiplano (12° S–18° S) shows that there is a localized high-speed anomaly that extends from the bottom of the crust to depths of ~ 120 km under the Altiplano basin and is surrounded by lower-speed anomalies on both western arc-forearc region and parts of the eastern Cordillera-sub-Andes^18^. Figure 4a shows the approximate fit of the geodynamic configuration of Model 1 (i.e., 4.31 Myrs-*model time* corresponding to ~ 10 Ma in *geological time*) to the seismic tomography model—across an E–W profile—where a piece of the lithospheric drip resides under the Altiplano crust and entraining into the mantle. On the other hand, there is a circulation of hot-buoyant asthenospheric mantle underneath the West and East Cordilleras at relatively shallower depths (< 150 km).

Further, the high-speed sub-crustal (Altiplano) anomaly is also correlated with thicker and lower density crust than areas to the east or west; however, it is also associated with lower elevations of the Altiplano such as the Lake Titicaca region. Ward et al.^18^ compared topography of the Central Andean Plateau to the elevation of an isostatically balanced reference crustal column and suggest that the area underlain by the positive sub-crustal anomaly is correlated with − 1.5 to − 2 km topographic residuals (i.e., elevations lower than predicted by Airy isostatic equilibrium). On the other hand, there are positive topographic residuals (i.e., elevations higher than predicted by Airy isostatic equilibrium) of up to 1.5 km on both western and eastern sides of this structure associated with negative residual topographic residuals. This is consistent with the double-humped/axisymmetric topographic expression and anticorrelation to crustal thickness predicted in the geodynamic modeling results presented above (Fig. 4a) where sinking lithospheric drip drives crustal flow from the sides and results in thicker crust above it.

Rapid surface uplift and the generation of multiple km of elevation between 25 and 10 Ma in the Central Andes has been documented by Δ47 clumped isotopes and δ^18^O analysis of soil carbonates, pollen assemblages and leaf wax lipids^16,61^, in addition to the already moderate elevation (i.e., 1.5–2 km) suggested for the late Oligocene to Miocene based on leaf physiognomy^62^ and phylogenetics^15^, fossil flora^63^, and structural reconstruction of regionally extensive ignimbrites along the Western Cordillera and western Central Andean Pacific slope^64^. In finer detail, Saylor and Horton^17^ suggested the attainment of high topography in the northern Central Andean region may have taken place in a non-uniform fashion^38,59^, wherein the Western Cordillera experienced faster and earlier surface uplift than the neighboring Altiplano region. Building on this finding, Sundell et al.^14^ shows that in addition to the Western Cordillera, the Eastern Cordillera also experienced earlier surface uplift than Altiplano. Both of these interpretations are consistent with the double humped geometry of the numerical modeling results controlled by lithospheric drip dynamics.

To aid in the comparison between the model predictions and the available uplift record of the Central Andes, we show a digram including the Model 1’s topographic evolution in central region (x = 1000 km) just above the lithospheric downwelling vs the paleoelevation history of the Altiplano region where the lithospheric dripping has operated (Fig. 4b,c). Here, both curves show that there is a rapid phase of surface uplift following the relatively subdued stage of elevation change. Although there are significant agreements between the results and observations in terms of the pattern and the fast increase of surface elevation, the time and the amount of uplift does not manifest an exact match. For instance, based on the paleoelevation estimates the rapid surface uplift in the Altiplano region is attained ~ 5 Myrs (15–10 Ma) whereas in the models this corresponds to ~ 1 Myr. In terms of the amount, the model predicts uplift of ~ 1–1.2 km (including the initial phase of subsidence) in 4.31 Myrs (and 1.5 km in 5 Myrs) whereas the uplift amount in such fast topographic growth is ~ 2 km between 15 and 10 Ma ^14^. Such offset arises due to the nature of the experimental work where there are assumed uncertainities in the starting conditions of the numerical model, for instance, the approximate lithospheric and crustal structure of Altiplano 15 Ma, size-available buoyancy of the instability, the exact viscosity of the dripping lithosphere.

Further, geodynamic models presented above do not account for all geological processes involved in the Cenozoic construction of plateau in the Central Andeas. Therefore, the surface uplift estimates may be less than that of invoked by paleoaltimetry studies (see above for Altiplano comparison). Such geodynamic processes include subduction induced flow following the removal of flat slab and crustal wedge tectonics^30^, and magmatic underplatings^65^. In addition to this, topographic evolution of the Central Andes during Cenozoic is also associated with considerable differences in terms of time and the amount both perpendicular and across the strike of the plateau in which the uncertainities of paleoelevation estimates are in the range of ~ 500 m or even more. Despite this, the lithospheric (drip) model results presented in this work are consistent with variable thicknesses of the crust and mantle lithosphere as measured by geophysical methods in the modern, as well as the double humped (rapid) surface uplift history as predicted by paleoelevation studies, which is subtly maintained in the modern with a relatively lower Altiplano adjacent higher cordilleras (Fig. 1). Regardless of the model parameter variation (i.e., faster convergence rate or thinner initial crustal thickness), such topographic expression is a salient feature of the geodynamic model prediction. Our results suggest a more nuanced development of Earth’s modern and ancient orogenic plateaus formed by Cordilleran orogenesis. In particular, the apparent discrepancy in crustal shortening and resultant crustal thickness, as demonstrated by the numerical experiments presented here, and in independent geologic studies^22,31–33^, have implications for studies of orogenic plateau evolution in deep time. Specifically, thickened crust as a result of crustal shortening may be partly decoupled from surface uplift of a region after certain time due to the removal of dense lower crust and mantle lithosphere, again, as seen in the modern anticorrelation between crustal thickness and surface elevation in the modern northern central Andes (Fig. 1c).

Linking lithospheric downwelling to crustal tectonics, our models show that crustal flow is controlled by crust-mantle coupling, different from models of other orogenic belts where crust mantle decoupling process is favoured. For instance, unlike the geodynamic conditions of large-hot orogens mainly controlled by the collision tectonics and thermo-mechanical models that consider initially two layer crust (upper-lower) that applies to channel flow hypothesis for the evolution of Himalaya-Tibet^66,67^, here, we considered a single crustal layer and only the smaller portion of the lower crust is dragged down by the lithospheric drip. Again, this prediction is in accord with the thicker crust above the faster wave speed anomaly (sub-Moho Altiplano anomaly). Over a set of numerical experiments, designed for understanding the enigmatic evolution of Arizaro (oval shaped) hinterland basin of Puna Plateau, Wang et al.^43^ showed that the decoupling between the crust and the dripping mantle lithosphere occurs with weaker lower crustal rheologies where topographic responses are rather less amplified than shown in this work. Because the rheological characteristics (varying strengths) and the thickness of the Andean crust may show differences across and normal to the strike of the orogenic chain, future, more regional 3-D geodynamic models will shed light upon the presence of potential partial melting in middle-lower crust of southern Peru recently inferred from magnetotelluric studies^68^.

This work primarily concentrates on the localized topographic effects of lithospheric instabilities (i.e., lower topography of Altiplano with respet to the sides) on the hinterland plate/behind arc region where relatively smaller wavelengths of residual topography estimates are aimed to explain. Models of subduction induced dynamic topography calculations are associated with the longer wavelength of the residual (possibly dynamic) topography estimates and may not account for such localized geological processes.

The lithospheric drip hypothesis postulated here does not necessarily preclude the lithospheric delamination in which the peeling off of the subducting flat slab or sub-arc mantle lithosphere from the overlying crust which has also been suggested to account for a variety geological and geophysical observations in Central Andes^44,58,69^. The onset of dripping (~ 15 Ma) associated with relatively *axisymmetric* pattern of geological and geophysical anomalies discussed here may have developed following to delamination process. Note that delamination (mobile lithosphere)^70–74^ in general rather reflects to larger amount of lithospheric removal and migrating waves of topographic anomalies anomalies, (*asymmetrical*) which may be harder to reconcile with the available seismological and geological observations of southern Peru region.

Overall, the results of this work suggest that lithospheric drips and induced crustal flow can explain enigmatic cause of rapid topographic uplift, basin formation, and the paradoxic relationship between the relatively thicker crust underlain by lower surface topography in central Andes and that lithospheric drips play a pivotal role in the evolution of Cordilleran orogenic cycle^39^.

## Methods

### Numerical modeling procedure

Numerical calculations are carried out by the SOPALE numerical software that uses arbitrary Lagrangian-Eularian finite element techniques to solve for the plane strain deformation of complex viscoplastic behavior of materials^75^. This technique is useful for treating finite deformations, and for tracking boundaries such as free surface and motion of internal particles at any depth and readers are referred to following studies for further explanations on how numerical (governing equations) calculations are conducted^52,75–77^.

### Rheological parameters

For rheological calculations we use laboratory measurements based on a viscous flow law of $\dot{\varepsilon } = A\sigma^{n} \exp \left( {\frac{ - Q}{{RT}}} \right)$. Here, $\dot{\varepsilon }$ is the strain rate, T is temperature, σ is deviatoric stress, and the variables A, n, Q and R are the viscosity parameter, power law exponent, activation energy, and ideal gas constant, respectively. For continental crust A = 1.1 × 10^–4^ MPa^−4^/s, n = 4, and Q = 223 kJ/mol are used, based on wet quartzite^46^. All numerical models use the viscous flow law with parameters Q = 0 and A = 10^−38^ Pa^−n^/s. Based on strain rates of $\dot{\varepsilon }$, 10^–12^ to 10^–17^ 1/s that are characteristic of flow in the models, this results in sub-arc mantle lithosphere lithosphere viscosity ranging from μ = 5.10^19^–5.10^22^ Pa s.

In the viscoplastic numerical model the deviatoric stress is determined at each computational node as the lesser value of either a yield stress, or viscous stress. For the frictional plastic yield stress a Drucker-Prager yield criterion is used, which is equivalent to the Coulomb criterion in plane strain.

### Model design, boundary conditions and thermal field

The initial 160 km thick lithosphere is made up of 55 km orogenically thickened crust (ρ_*o*_ = 2840 kg m^−3^, blue) and 105 km thick sub-arc mantle lithosphere (ρ_*o*_ = 3300 kg m^−3^, yellow) overlying a sub-lithospheric (asthenospheric) mantle region (ρ_*o*_ = 3260 kg m^−3^, orange). Such initial lithospheric thickness (Central Andes) approximates to 120–150 km boundary for the base of the lithosphere in the Cordilleran arc system (e.g., Altiplano), suggested by Heit et al.^78^ based on receiver function studies. In these models, the reference density of the sub-arc mantle lithosphere is initially set to be higher than the underlying asthenospheric mantle. Such Cordilleran-type arc roots are often anomalously dense owing to the presence of subduction derived melts and fluids^79–81^. The numerical (width) and (depth) resolution is 201 × 101 Eulerian nodes and 601 × 301 Lagrangian nodes. Half of the Eulerian and Lagrangian elements are concentrated in the top 160 km in order to enhance resolution in the lithosphere. We have extended the depth of the solution space into the lower mantle so that the sinking mantle lithosphere material moves away from the lithosphere. No material flux is allowed through the bottom boundary, but varying plate velocities are imposed on the left margin in the horizontal direction to approximate plate convergence (e.g., *VP* = 1–3 cm year^−1^). Erosion and deposition processes are not included in the experiments. The model has a free top surface, allowing topography to develop as the model evolves. The mechanical boundary conditions at the other three sides are defined by zero tangential stress and normal velocity (e.g., “free slip”). In these models, density, ρ, is a function of composition and temperature, ρ = ρ_o_ (1 − α (T − T_o_)), where T is temperature, α = 2 × 10^−5^ K^−1^ is the coefficient of thermal expansion, T_o_ = 25 °C is the reference temperature, and ρ_o_ is the reference density that depends on material. Experiments showed that an increase in the thermal expansion coefficient (e.g., α = 3 × 10^−5^ K^−1^) has relatively minor effect on these lithospheric scale model calculations. The initial geotherm for the experiments is laterally uniform and is defined by a surface temperature of 25 °C, an increase to 550 °C at the Moho, an increase to 1350 °C at the base of the mantle lithosphere, and an increase to 1525 °C at the bottom of the model.

We note that 2-D numerical modeling conducted in this work only captures cross section of the lithospheric dynamics and the associated surface response to it. Nevertheless this provides information on the first order characteristics of rapid evolution of orogenesis in southern Peru. Such effort was made for two purposes. Firstly, 2D numerical experiments so far have been more successful in capturing the crust-lithospheric scale geological evolution of the orogens by higher resolution numerical modeling implementation. Secondly, as shown in Fig. 1, the “sub-moho Altiplano anomaly” is much narrower in the north and it gradually extends to the south towards both western-eastern Cordilleras. In that case, our model applies to the central cross section (above lake Titicaca) where the reconciliation between the model predictions vs observations are made carefully. For instance across that profile, seismic anomaly is clearly visible^18^ and available paleoaltimetry estimates suggest rapid surface uplift (between 15 and 10 Ma)^14^.

## Supplementary Information

Supplementary Figure 1.
