# Supplementary material for: Rapid surface uplift and crustal flow in the Central Andes (southern Peru) controlled by lithospheric drip dynamics
Source: Sci Rep. 2022 Apr 1;12:5500. doi: 10.1038/s41598-022-08629-8 (PMC8975996; doi:10.1038/s41598-022-08629-8)
Supplement: Supplementary file 1 — Supplementary Figure 1. [file 41598_2022_8629_MOESM1_ESM.pdf]

Supplementary Figure 1

3.55 Myrs

Viscosity

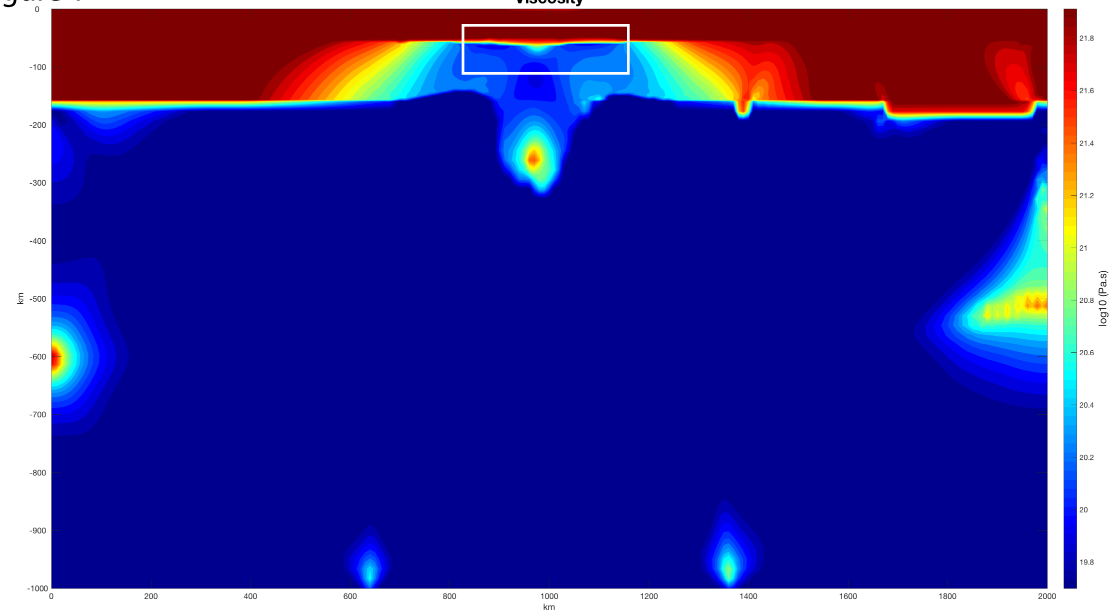

4.05 Myrs

Viscosity

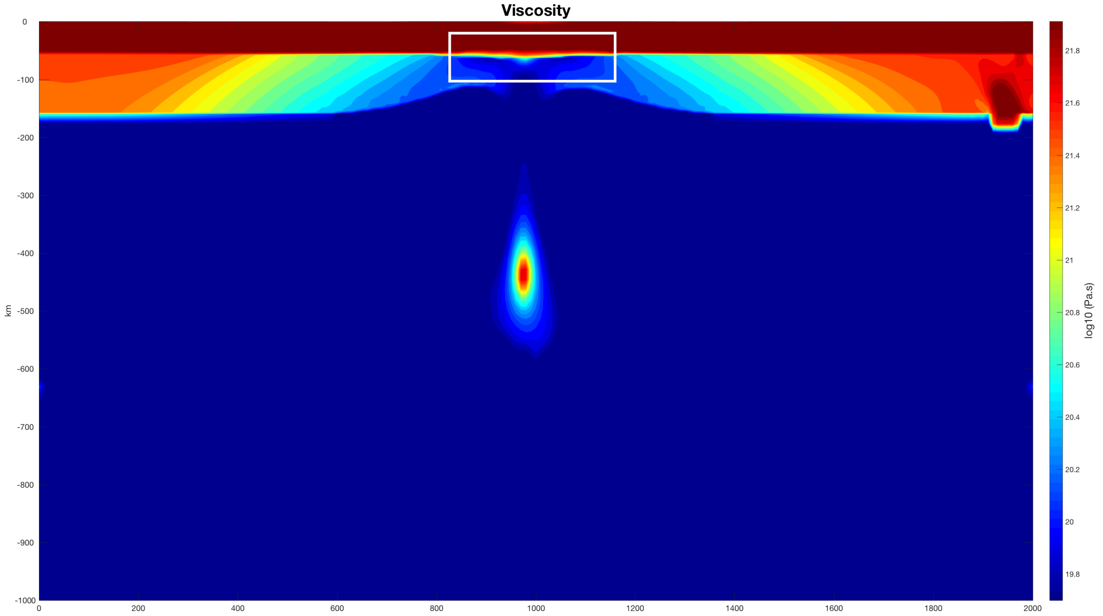

4.31 Myrs

Viscosity

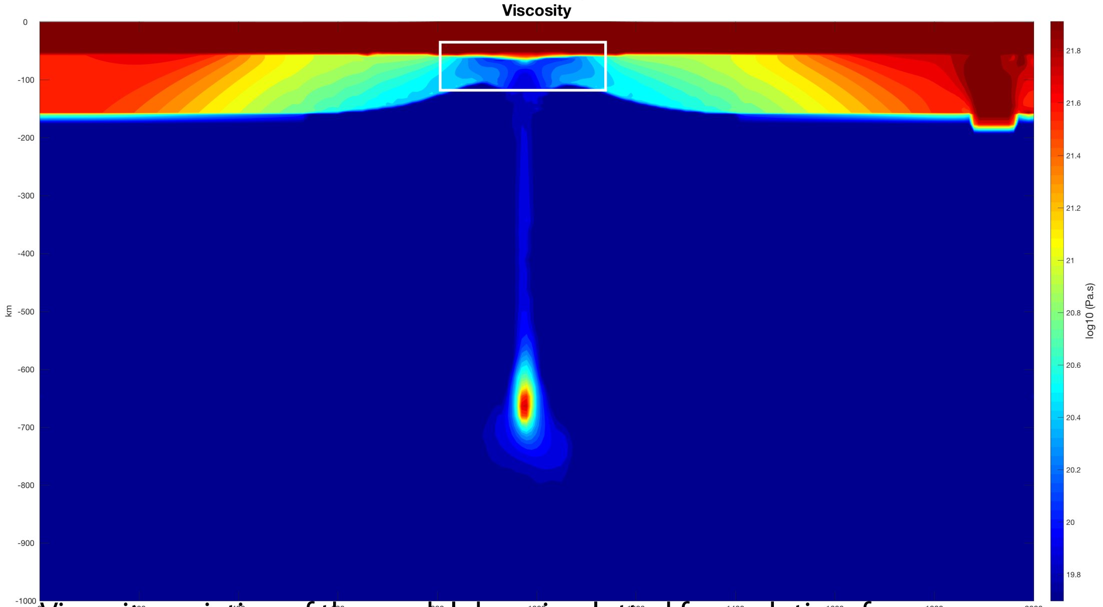

Viscosity variation of the model domain plotted for each time frame.
Note the relatively higher viscosity (shown by rectangular frame) in the central part of the Moho.
This suggests the entrainment (flow) of the crust into the mantle lithosphere downwelling.
Figure is created by Matlab software version R2016b (https://www.mathworks.com/).
